# The association between exercise, activities, and frailty in older Chinese adults: a cross-sectional study based on the Chinese Longitudinal Healthy Longevity Survey (CLHLS) data

**DOI:** 10.1186/s12877-025-05802-2

**Published:** 2025-02-26

**Authors:** Linyan Dai, Yangyang Tang, Yihong Guo, Xia Lai, Xingsheng Wang, Baoshan Li

**Affiliations:** Department of Geriatrics, Chongqing University Central Hospital, Chongqing Emergency Medical Center, Chongqing, China

**Keywords:** Frailty, Exercise/activities, The Chinese longitudinal healthy longevity survey (CLHLS), Cross-sectional study

## Abstract

**Objectives:**

The purpose of this study was to evaluate the association between exercise/activities and frailty in older Chinese adults using the Chinese Longitudinal Healthy Longevity Survey (CLHLS).

**Methods:**

This cross-sectional study included 6862 participants aged 65 years or older from the CLHLS 2018. Frailty was assessed using a 38-variable frailty index (FI). Activities included Tai chi, square dance, garden work, raising animals/pets, playing cards/mah-jongg, social activity and housework. Multivariate logistic regression was used to assess the association between exercise/activities and frailty, adjusting for covariates such as gender, age, BMI, chronic diseases, residence, education, co-residence, economic status, smoking, drinking, physical labor history, and nutritional supplements.

**Results:**

The mean age of this study was 81.5 (SD = 10.3)years, with 3567 (52%) females. Frailty prevalence was 32.3%. The prevalence of exercise and daily activities was 31% and 78.6%, respectively. Multivariate logistic regression analysis showed that the likelihood of frailty increased with decreased exercise (OR = 1.85, 95% CI: 1.57, 2.18, *P* < 0.001) and activities (OR = 4.43, 95% CI: 3.74, 5.25, *P* < 0.001).

**Conclusions:**

Participation in exercise/activities is associated with a lower likelihood of frailty. Regular housework may also contribute to reducing frailty risk.

**Supplementary Information:**

The online version contains supplementary material available at 10.1186/s12877-025-05802-2.

## Introduction

Frailty, a complex geriatric syndrome characterized by decreased physiological reserve and increased vulnerability to stressors, has emerged as a significant burden with the global aging population [1]. Aging leads to a decline in cellular homeostasis due to the loss of free radicals’ signaling function in cell metabolism, reducing cellular resilience and the body’s ability to withstand stress [2]. This results in heightened susceptibility to low-power stressors and adverse outcomes such as disability, hospitalization, and institutionalization [3]. Rockwood’s Frailty Index, based on cumulative health deficits, assesses multiple aspects including diseases, physical and cognitive functions, and psychological states [4].The index score increases with the number of health problems, reflecting greater frailty. Frailty is potentially preventable, and physical exercise is considered one of the most effective interventions to delay and treat frailty [5–9].

Exercise has been associated with numerous benefits, including improved cardiovascular function [10], enhanced muscle strength [11], and better mental well-being [12]. To combat the occurrence and development of frailty, various types of exercise can be categorized into aerobic exercises (e.g., walking, swimming), resistance training (e.g., weightlifting), flexibility exercises (e.g., yoga), and balance exercises (e.g., Tai Chi). Among these, resistance training has shown the best potential to reduce frailty in older adults [13].Community-based exercise interventions involve structured physical activity programs conducted in community settings, such as group exercises in community centers, outdoor activities, or home-based programs. These interventions are designed to be accessible and sustainable for older adults. A recent meta-analysis of 22 randomized controlled trials involving 1815 participants demonstrated that community-based exercise interventions are superior to minimal intervention (less-structured or unstructured activities) for improving health status in pre-frail older adults [14].

While previous studies have demonstrated the benefits of exercise in reversing frailty, this study uniquely evaluates the association between various types of physical activities and frailty in a large, nationally representative sample of older Chinese adults. Additionally, it explores the impact of different frequencies and types of activities on frailty, providing more nuanced insights into the role of physical activity in frailty prevention. We hypothesized that older adults, those with no exercise/activity participation, would exhibit a significantly higher likelihood of being frail. To test the hypothesis, we assess the association between exercise/activities and frailty in this population using the CLHLS.

## Methods

### Study data

The study utilized data from the Chinese Longitudinal Healthy Longevity Survey (CLHLS) conducted in 2018, a national survey exploring the impact of health-related factors on aging outcomes among Chinese individuals. The survey included participants aged 65 years or older from over 500 sample areas across 22 provinces, municipalities, and autonomous regions. Data collected encompassed demographic information, social and economic status, self-assessed health quality, exercise habits, cognitive function, depressive symptoms, anxiety, activities of daily living, chronic diseases, and medication use. Cognitive function, depressive symptoms, and anxiety were not included as confounding factors in this study because the primary focus was on the physical aspects of frailty. While these factors can influence overall health, their impact on frailty is more indirect and was not the primary focus of this analysis. The study aimed to assess the direct association between exercise/activities and frailty, controlling for factors that have a more immediate and measurable impact on physical frailty.Detailed information about CLHLS has been published previously [15].

### Sample

Of the 2018 wave of CLHLS, we focused on the elderly aged 65 and above. We excluded those with missing data in the construction of the Frailty Index (FI).We also excluded the elderly with both visual and auditory impairments, paralysis and dementia. Elderly individuals with visual and auditory impairments, paralysis, and dementia were excluded from the study because these conditions can significantly affect the ability to perform physical activities and may confound the association between exercise/activities and frailty. Including these individuals could have introduced additional variability and complexity, making it difficult to isolate the effects of exercise/activities on frailty. However, future studies could explore the impact of exercise/activities on frailty in these specific populations. A total of 6862 older adults were included for analysis.

### Data definitions

#### Frailty

Both the physical frailty phenotype and the cumulative health deficit index are used to define frailty [16].The frailty index used in this study has been validated in various populations, including older adults in the Italy [17], United States [18]. These studies have demonstrated the reliability and validity of the frailty index in assessing frailty in older adults. In our study, we used the frailty index based on cumulative health deficits due to the availability of comprehensive health data in the CLHLS. Fried’s phenotype model was not used because the necessary data for this model, such as grip strength and gait speed, were not available in the CLHLS dataset., A 38-item frailty index (FI) was created based on a validated and published method. (See APPENDIX for the details) These items were chosen as they were all relevant to health status and tended to become more prevalent with age but were not fully universal in older people. Most of the items had a binary option and were scored as 0 (absence) or 1 (present). To the items with ordinal options, e.g., always, often, sometimes, seldom, and rarely or never, we assigned 0, 0.25, 0.5, 0.75, and 1 to them respectively. The FI score, which ranged from 0 to 1, was calculated for each responder as the total of the item scores divided by the number of items they possessed. Participants were disqualified if they possessed fewer than 30 items. After calculating the FI score, we used the following cutoff points from a previous study to classify participants as non-frail (FI ≤ 0.25) and frail (FI > 0.25).

#### Exercises/activities

The exercise/activity variables were categorized based on the questionnaire items in the CLHLS.The exercise we defined refers to purposeful fitness activities, corresponding to D9-1 in the questionnaire: Do you often exercise now? (Referring to purposeful fitness activities, such as walking, playing ball games, running, qigong, etc.). The activities we defined refer to physical activities in daily life, which are not purposeful but rather daily behaviors, included tai chi, square dance, garden work, raising domestic animals/pet, playing cards/mah-jongg, social activity and housework, corresponding to D11 in the questionnaire: Do you currently engage in the following activities?

Self-reported activities may be subject to recall bias, as participants may not accurately remember their activity levels. To mitigate this bias, the study used a structured questionnaire with clear and specific questions about activity types and frequencies. Additionally, the study included multiple questions to cross-validate the reported activities. However, some recall bias may still exist, and this is acknowledged as a limitation of the study.

#### Covariates

Demographic characteristics, socioeconomic status, and health-related variables were included as covariates, including gender, age, BMI(Body Mass Index), chronic diseases (diabetes, heart disease, and stroke: yes or no), residence (city, town and rural), years of schooling (0, 1 to 6, 7 to 9, 10 to 12 and at least 13), co-residence (house member(s), alone and in situation), perceived economic situation (rate their economic status in compared with that of other local people: very rich, rich, so-so, poor and very poor). Regarding lifestyle behaviors, we extracted smoking, drinking, physical labor before, nutritional supplements, all these lifestyle behaviors were categorized as yes versus no.

### Statistical analysis

IBM SPSS 25.0 statistical software was used for data analysis. Categorical data was presented with frequencies and percentages. Continuous data was presented with means and standard deviations. Comparisons between the frailty and non-frailty groups were made using the Chi-square test for categorical variables and the t-test for continuous variables. Univariate logistic regression was adopted to identify the factors associated with frailty status using the crude OR. Potentially confounding factors were based on factors related to frailty; exercise/activities were the independent variable, and the frailty status was the dependent variable. Multiple logistic regression was employed to identify the independent association between exercise/activities and frailty after adjusting for potential confounding factors [19]. We also conducted subgroup analyses on different types of activities, activity frequencies (two groups were set, the control group is those with a frequency more than once a week), and the number of activities carried out simultaneously. Furthermore, stratified analyses were performed based on gender. A P-value less than 0.05 was considered statistically significant.

## Results

### Baseline characteristics

A total of 6862 subjects, with an average age of 81.5 (SD = 10.3) were included in this study, comprising 3567 females (52%) and 3295 males (48%). There were 2213 cases (32.3%) in the frail stage and 4649 cases (67.7%) in the non-frail stage. The majority of the total sample were living with house member (s) (79.2%). The proportion of the non-frail group engaging in exercise stands at 36.4%, while that of the frail group is 19.7%. In terms of taking physical activity, 90% of the non - frail group are active, compared to 54.8% in the frail group. In the non -frail group, regarding the chronic diseases, the prevalence rates of diabetes, heart disease and stroke or cerebrovascular diseases (CVD) were 9.8%, 15.8% and 9.4%, respectively. In terms of lifestyle behavior, 20.2% smoked, and 19.3% drank. There were statistically significant differences between the two groups in residence, economic status, BMI, the use of nutrient supplements, heart disease, stroke or cardiovascular disease (CVD) and the situation of previous physical labor (*P* < 0.05). There was no significant difference in the prevalence of diabetes between the two groups. In addition, we found that the proportions of smokers and drinkers were relatively higher in the non-frail group (Supplementary Table S1).

### Univariate analysis between independent factors and frailty

Compared to older adults with non-frailty, frailty individuals were more likely to be female, older, illiterate (years of schooling (years), *n* = 0), living in an institution, with heart disease, with stroke or CVD, and less likely to take part in exercise and activities (Table 1). 

**Table 1:** Univariate analysis between independent variable and frailty

Variable	Statistics	Frailty OR(95%CI)	*P* value
**Gender, n (%)**			
Male	3295 (48)	Reference	
Female	3567 (52)	1.77 (1.60–1.96)	< 0.001
**Age (years)**	81.5 ± 10.3	1.11 (1.10–1.11)	< 0.001
**Residence**,** n (%)**			
city	1622 (23.6)	Reference	
Town	2400 (35)	0.89 (0.78–1.02)	0.090
Rural	2840 (41.4)	0.85 (0.75–0.97)	0.013
**Years of schooling (years)**,** n (%)**			
0	2425 (35.3)	Reference	
1–6	2389 (34.8)	0.42 (0.37–0.47)	< 0.001
7–9	726 (10.6)	0.34 (0.28–0.41)	< 0.001
10–12	384 (5.6)	0.29 (0.22–0.38)	< 0.001
At lest 13	209 (3)	0.57 (0.42–0.77)	< 0.001
**Co-residence**,** n (%)**			
Living with house member(s)	5436 (79.2)	Reference	
Living alone	1160 (16.9)	1.02 (0.89–1.17)	0.767
In an institution	193 (2.8)	3.89 (2.89–5.25)	< 0.001
**Economic situation**,** n (%)**			
Very rich	130 (72.2)	Reference	
rich	843 (72.8)	0.97 (0.68–1.38)	0.872
So so	3250 (68.6)	1.19 (0.86–1.66)	0.301
poor	353 (55.2)	2.11 (1.47–3.03)	< 0.001
Very poor	42 (47.7)	2.85 (1.66–4.84)	< 0.001
**Smoking**,** n (%)**			
Yes	1180 (17.4)	Reference	
No	5619 (82.6)	1.96 (1.69–2.28)	< 0.001
**Drinking**,** n (%)**			
Yes	1096 (16.2)	Reference	
No	5668 (83.8)	2.25 (1.92–2.64)	< 0.001
**BMI (kg/m**^**2**^)	23.0 ± 8.2	0.98 (0.97–0.99)	0.001
**Diabetes**,** n (%)**			
Yes	699 (10.2)	Reference	
No	6163 (89.8)	0.88 (0.74–1.03)	0.113
**Heart disease**,** n (%)**			
Yes	1169 (17)	Reference	
No	5693 (83)	0.77 (0.67–0.87)	< 0.001
**Stroke or CVD**,** n (%)**			
Yes	692 (10.1)	Reference	
No	6170 (89.9)	0.80 (0.68–0.94)	0.006
**Nutrient supplements**,** n (%)**			
YES	892 (13.2)	Reference	
NO	5880 (86.8)	0.80 (0.69–0.93)	0.003
**Physical labor before**,** n (%)**			
YES	5075 (76.4)	Reference	
NO	1570 (22.9)	1.20 (1.06–1.35)	0.003
**Exercise**,** n (%)**			
YES	2127(31)	Reference	
NO	4735(69)	2.32 (2.06–2.62)	< 0.001
**Activities**,** n (%)**			
YES	5396(78.6)	Reference	
NO	1466(21.4)	7.40 (6.52–8.40)	< 0.001

### Multiple logistic regression between exercise/activities and frailty

The results of the multiple logistic regression analysis for different adjustments are shown in Table 2. There was a significant association between exercise (OR = 1.97, 95% CI:1.73, 2.24, *P* < 0.001)/activities (OR = 6.94, 95% CI: 6.10, 7.89, *P* < 0.001) and the likelihood of frailty in the unadjusted model. After adjusting for potential covariates, the association still existed. Compared to older adults with exercise/activities, individuals with no exercise had a higher likelihood of frailty (OR = 1.85, 95% CI: 1.57, 2.18, *P* < 0.001), and individuals with no activities also had a higher likelihood of frailty (OR = 4.43, 95% CI: 3.74, 5.25, *P* < 0.001). Among them, female gender, advanced age, living in institutions, poverty, higher BMI, cardiovascular diseases, and taking nutritional supplements are likely risk factors for frailty, while living in rural areas, receiving education, and alcohol consumption may be protective factors. The Supplementary Table S2 presented multiple logistic regression between exercise/activities and frailty based on gender. Regardless of gender, the association between the above-mentioned exercise/activities and frailty still existed.

**Table 2:** The association between exercise/activities and frailty, controlling for confounding factors

	Frailty OR(95%CI)	*P* value	Adjusted frailty OR(95%CI)	*P* value
**Exercise**				
YES	Reference		Reference	
NO	1.97 (1.73–2.24)	< 0.001	1.85(1.57-2.18)	< 0.001
**Activities**				
YES	Reference		Reference	
NO	6.94(6.10–7.89)	< 0.001	4.43(3.74-5.25)	< 0.001
**Gender**				
Male			Reference	
Female			1.57(1.33-1.86)	< 0.001
**Age (years)**			1.09(1.08-1.10)	< 0.001
**Residence**				
city			Reference	
Town			0.78(0.64-0.96)	0.019
Rural			0.73(0.59-0.89)	0.002
**Years of schooling (years)**				
0			Reference	
1–6			0.78(0.66-0.92)	0.003
7–9			0.86(0.66-1.12)	0.262
10–12			0.69(0.48-0.99)	0.045
At lest 13			1.18(0.77-1.81)	0.442
**Co-residence**				
Living with housemember(s)			Reference	
Living alone			0.91(0.76-1.09)	0.311
In an institution			1.63(1.08-2.46)	0.021
**Economic situation**				
Very rich			Reference	
rich			0.78(0.49-1.24)	0.296
So so			1.20(0.78-1.87)	0.411
poor			2.91(1.79-4.74)	< 0.001
Very poor			2.87(1.47-5.60)	0.002
**Smoking**				
Yes			Reference	
No			1.08(0.87-1.33)	0.491
**Drinking**				
Yes			Reference	
No			1.55(1.24-1.93)	< 0.001
**BMI (kg/m**^**2**^)			1.01(1.00-1.02)	0.015
**Heart disease**				
Yes			Reference	
No			0.76(0.63-0.91)	0.003
**Stroke or CVD**				
Yes			Reference	
No			0.81(0.65-1.01)	0.063
**Nutrient supplements**				
YES			Reference	
NO			0.62(0.51-0.76)	< 0.001
**Physical labor before**				
YES			Reference	
NO			1.13(0.95-1.36)	0.171

### Association between activities and frailty

**Fig. 1:**
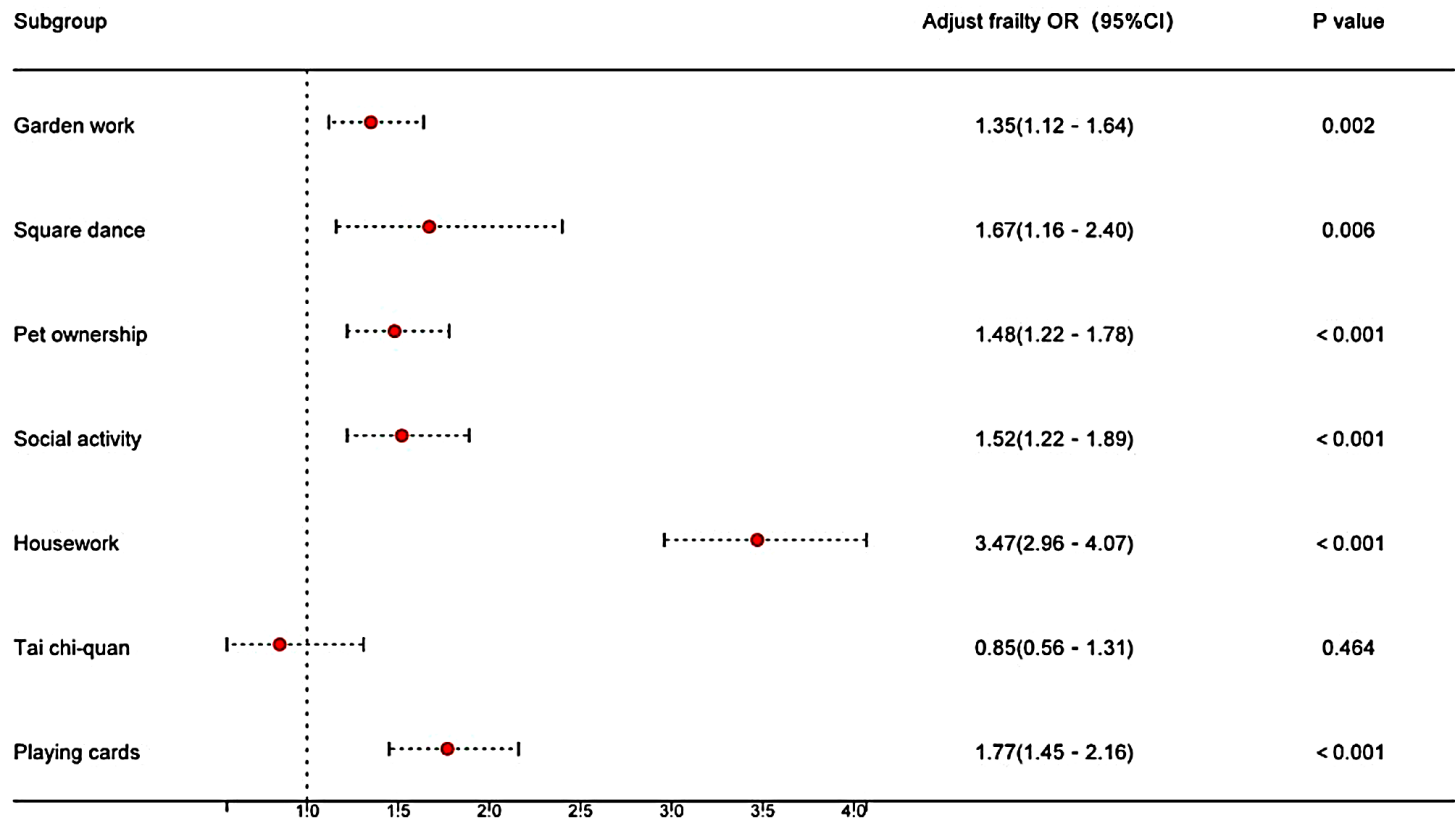
The association between activities and frailty

After adjusted potential variables, older adults who engaged in physical activities such as square dancing, gardening, pet ownership, playing cards, socializing, and housework were less likely to be frail compared to those who did not engage in these activities. Housework was found to have the strongest association with lower likelihood of frailty (OR = 3.47, 95% CI: 2.96, 4.07, *P* < 0.001) (Fig. 1, Supplementary table S3). Compared with individuals who engage in activities like square dancing, gardening, pet ownership, playing cards, and housework at least once a week, those with less frequent participation in activities have a greater likelihood of frailty (Supplementary table S4). Compared to older adults who did not engage in any activities (reference group), those who engaged in four different types of activities simultaneously had a significantly lower likelihood of frailty (OR = 0.11, 95% CI:0.07, 0.17, *P* < 0.001). The benefits of engaging in multiple activities were significant up to four activities. While the adjusted frailty OR remains statistically significant when five activities are performed, the magnitude of the effect decreases, indicating that the benefits may plateau or diminish with more than four activities. (Supplementary table S5).

Smoking and alcohol consumption were not included as potential confounders in the adjusted model because the primary focus of the study was on the association between physical activity and frailty. While smoking and alcohol consumption are associated with frailty, their inclusion would have added complexity to the model without significantly altering the results. However, future studies could explore the combined effects of physical activity, smoking, and alcohol consumption on frailty.

## Discussion

Our study demonstrated that participation in exercise and daily activities is associated with a lower likelihood of frailty. Older adults who engaged in activities such as square dancing, gardening, pet ownership, playing cards, socializing, and housework had a reduced risk of frailty compared to those who did not participate in these activities. These findings highlight the importance of promoting exercise and daily activities among older adults to mitigate frailty risk.

Our research findings found that elderly men who were previously engaged in physical activities have a lower likelihood of frailty, while the protective effect is not as significant among elderly women. Men usually have more muscle mass and greater muscle strength than women when they are young [20]. Elderly men who were previously involved in physical activities, due to their better initial muscle reserves, may still retain relatively more muscle mass and better muscle strength even when their muscles have decreased with age. The testosterone level in men’s bodies is relatively high, and testosterone plays an important role in maintaining muscle mass, bone density, and the body’s metabolic functions [21]. However, after menopause, women experience a significant decline in estrogen levels, which may lead to a series of physiological changes such as reduced bone density, decreased muscle mass, and increased fat, thereby increasing the risk of frailty [22, 23]. In terms of behavioral and social factors, women are often family caregivers, with their energy divided and thus having insufficient exercise. Men, on the other hand, are more involved in socio - economic activities and may have more opportunities for exercise at work. In addition, men are more likely to engage in higher intensity physical labor. Moderate physical labor is beneficial to physical functions. However, if there is long - term excessive wear and tear, they are prone to musculoskeletal problems in old age, which instead increases the risk of frailty. Women, on the other hand, are mostly engaged in light - physical or service - oriented jobs, sitting for long periods with little movement. They also face the problem of declining physical functions, thus increasing the risk of frailty. In addition, factors such as being female, being of a higher age, having a low body mass index (BMI), living in urban areas, having a lower education level, living in institutions, having a lower economic level, and having a history of chronic diseases all increase the likelihood of frailty, which is generally like the results of previous related studies [1].

We found that engaging in a wide range of daily activities may associate with a reduced frailty risk. They may achieve their protection against frailty in the following aspects. Long-term dancing can not only increase muscle strength and endurance but also improve the body’s coordination and flexibility, making the elderly’s bodies more stable and reducing the risk of falls [24]. Loneliness can suppress the immune system’s function and lead to a decline in brain function. Alleviating loneliness helps maintain the body’s normal physiological functions [25]. Interacting with pets can make the human body secrete beneficial substances such as endorphins, which help relax vascular smooth muscles, lower blood pressure, and relieve the burden on the heart [26]. Rich social relationships can promote the body to secrete hormones such as oxytocin and endorphins. These substances have anti-inflammatory effects and can enhance the function of the human immune system, enabling the body to better resist diseases and inflammation [27]. Social interaction requires the participation of the brain, such as communication, thinking, and memory, which can stimulate the activity of brain nerve cells, promote the release of neurotransmitters, enhance the brain’s plasticity, delay the occurrence of neurodegenerative diseases, and reduce the risk of cognitive decline and dementia. Housework activities like cleaning and tidying up require certain cognitive abilities and attention. Regularly doing housework can stimulate the brain’s nerve cells, improve the brain’s flexibility and reaction speed, and help prevent cognitive decline. A study published by a Singapore research team pointed out that elderly people who often do housework have higher scores in cognitive function and attention [28]. Housework involves various body movements such as bending, stretching, and carrying, which can exercise all parts of the body, enhance muscle strength, joint flexibility, and body balance ability, reduce the risk of falls, and prevent the decline of physical functions [29]. When playing cards, people need to concentrate, think, remember, and make decisions, which can expand the blood flow area in the brain, promote blood circulation, provide sufficient oxygen and nutrients for brain cells, maintain the normal metabolism of brain cells, delay brain aging, and reduce the risk of frailty caused by brain diseases [30]. Playing cards is usually a multi-person activity. During the process, people can communicate and interact, enhance their feelings for each other, relieve loneliness, and obtain emotional support. Gardening activities allow the elderly to be in a natural environment, in contact with flowers, plants, trees, and sunlight, which can stimulate the human senses, help people relax, relieve stress and anxiety [31],. Gardening is a comprehensive full-body exercise that can enhance muscle strength, joint flexibility, and body balance ability, prevent the decline of physical functions. Our research did not identify a correlation between Taijiquan and frailty. This outcome could be attributed to the relatively small sample size, which limits the statistical power to detect a relationship, and the challenge of fully controlling confounding factors. Additionally, compared with the aforementioned daily activities, the lower volume of physical activity associated with Taijiquan practice might lead to a diminished protective effect against frailty. Nevertheless, existing literature indicates that Taijiquan, as a structured physical activity, has the potential to enhance balance, muscle strength, and cardiovascular function. Studies [4] have demonstrated the positive impacts of regular Taijiquan practice on these aspects, suggesting that, under appropriate conditions, it can be a beneficial form of exercise for overall health, although this was not reflected in the current study’s findings regarding the relationship with frailty.

We observed that the elderly who engaged in daily activities more than once a week were less likely to be frail compared to those with lower frequencies of activities. Studies [4, 32] have shown that regular exercise with a higher frequency has a positive impact on muscle adaptation among the elderly. The elderly who do not exercise for a long time or exercise very few times a week have a significantly increased risk of developing diabetes and obesity [33]. Metabolic disorders will affect the normal functions of various organs in the body. For example, insulin resistance will affect the uptake and utilization of glucose by cells, resulting in insufficient energy supply and a decline in physical function [34]. In addition, obesity will increase the burden on joints and limit physical activities.

A study pointed out that after undergoing a 12-week progressive moderate-intensity resistance training program, with three sessions per week and an intensity of 60% 1RM, the muscle strength and muscle mass of the elderly were significantly improved, indicating that moderate-intensity resistance training can effectively increase the muscle mass and strength of the elderly while reducing oxidative stress [35]. However, our research also found that the benefits decreased when five activities were carried out simultaneously. From the perspective of the overuse of remaining muscles, the muscles of the elderly themselves have already experienced a certain degree of atrophy and functional decline with age, and the remaining muscle strength and endurance are relatively weak.,Excessive exercise makes the muscles bear a load that exceeds their capacity, easily leading to muscle strains or even tears, increasing the risk of falls and injuries [36]. Excessive exercise may also have an inhibitory effect on the immune system of the elderly. The previous study has shown that high-intensity and long-duration exercise may lead to an unstable state of the immune function of the elderly in the short term, increasing the possibility of pathogen invasion and infectious diseases, and thus may affect the occurrence and development of frailty [37].

The findings of this study have important policy implications. They suggest that promoting physical activity among older adults could be an effective strategy to reduce the prevalence of frailty. Public health initiatives and community programs should focus on encouraging older adults to engage in regular physical activities, such as square dancing, gardening, and housework. Additionally, healthcare providers should consider incorporating physical activity recommendations into routine care for older adults to promote healthy aging.

Despite the above findings, our study still has limitations. Firstly, this is a cross-sectional study, which only demonstrates associations rather than causal relationships. Therefore, the findings should be interpreted with caution, and further longitudinal studies are needed to establish causality.Secondly, this study has limitations regarding self-reported activity levels and cultural and sports preferences, which affect the generalizability of the research results to different populations or environments. Thirdly, the study did not strictly define the exercise intensity. This might have affected the accuracy of our results, as different exercise intensities can have different impacts on frailty. Future studies should incorporate objective measurements of exercise intensity, such as heart rate monitors or metabolic equivalents (METs). Participants could be stratified according to intensity levels to examine the different effects on the risk of frailty. It is also important to consider the potential for reverse causation, where frailty may lead to lower activity levels rather than the reverse. Frail older adults may be less likely to engage in physical activities due to physical limitations and reduced mobility. This bidirectional relationship highlights the need for further research to explore the causal mechanisms between physical activity and frailty. By addressing these limitations and conducting further research, we can gain a more comprehensive understanding of the relationship between exercise and frailty and develop more effective strategies to promote healthy aging.

## Conclusions

Participation in exercise and daily activities is associated with a lower likelihood of frailty in older Chinese adults. This study provides valuable insights into the potential benefits of various activities in reducing frailty risk. Future research should further investigate these findings and explore the underlying mechanisms to inform effective interventions for frailty prevention.

## Electronic supplementary material

Below is the link to the electronic supplementary material.

Supplementary Material 1

## Data Availability

Data is provided within the manuscript and supplementary information files.
