# Supplementary material for: The association between exercise, activities, and frailty in older Chinese adults: a cross-sectional study based on the Chinese Longitudinal Healthy Longevity Survey (CLHLS) data
Source: BMC Geriatr. 2025 Feb 26;25:131. doi: 10.1186/s12877-025-05802-2 (PMC11863621; doi:10.1186/s12877-025-05802-2)
Supplement: Supplementary file 1 — Supplementary Material 1 [file 12877_2025_5802_MOESM1_ESM.docx]

**Supplementary Table S1. Baseline of characteristics among total sample and frailty groups**

| **Variable** | **No frailty (n=4649)** | **Frailty (n=2213)** | **χ^2^/*t*** | **P value** |
| --- | --- | --- | --- | --- |
| **Gender, n (%)** |  | | | |
| Male | 2443 (52.5) | 852 (38.5) | 118.559a | <0.001 |
| Female | 2206 (47.5) | 1361 (61.5) |  |  |
| **Age (years)** | 78.5±9.1 | 87.8±9.7 | 38.134b | <0.001 |
| **Residence, n (%)** |  | | | |
| city | 1060 (22.8) | 562 (25.4) | 6.264a | 0.044 |
| Town | 1630 (35.1) | 770 (34.8) |  |  |
| Rural | 1959 (42.1) | 881 (39.8) |  |  |
| **Years of schooling (years), n (%)** |  | | | |
| 0 | 1349 (32.3) | 1076 (54.8) | 295.413a | <0.001 |
| 1-6 | 1795 (43) | 594 (30.3) |  |  |
| 7-9 | 571 (13.7) | 155 (7.9) |  |  |
| 10-12 | 312 (7.5) | 72 (3.7) |  |  |
| At lest 13 | 144 (3.5) | 65 (3.3) |  |  |
| **Co-residence, n (%)** |  | | | |
| Living with housemember(s) | 3745 (81.3) | 1691 (77.6) | 91.193a | <0.001 |
| Living alone | 794 (17.2) | 366 (16.8) |  |  |
| In an institution | 70 (1.5) | 123 (5.6) |  |  |
| **Economic situation, n (%)** |  | | | |
| Very rich | 130 (2.8) | 50 (2.3) | 79.356a | <0.001 |
| rich | 843 (18.3) | 315 (14.4) |  |  |
| So so | 3250 (70.4) | 1489 (68.1) |  |  |
| poor | 353 (7.6) | 287 (13.1) |  |  |
| Very poor | 42 (0.9) | 46 (2.1) |  |  |
| **Smoking, n (%)** |  | | | |
| Yes | 930 (20.2) | 250 (11.4) | 79.276a | <0.001 |
| No | 3680 (79.8) | 1939 (88.6) |  |  |
| **Drinking, n (%)** |  | | | |
| Yes | 886 (19.3) | 210 (9.6) | 102.274a | <0.001 |
| No | 3698 (80.7) | 1970 (90.4) |  |  |
| **BMI (kg/m^2^)** | 23.0±8.2 | 22.4±6.7 | 3.386a | 0.001 |
| **Diabetes, n (%)** |  | | | |
| Yes | 455 (9.8) | 244 (11) | 2.515a | 0.113 |
| No | 4194 (90.2) | 1969 (89) |  |  |
| **Heart disease, n (%)** |  | | | |
| Yes | 734 (15.8) | 435 (19.7) | 15.873a | <0.001 |
| No | 3915 (84.2) | 1778 (80.3) |  |  |
| **Stroke or CVD, n (%)** |  | | | |
| Yes | 437 (9.4) | 255 (11.5) | 7.452a | 0.006 |
| No | 4212 (90.6) | 1958 (88.5) |  |  |
| **Nutrient supplements, n (%)** |  | |  | |
| YES | 566 (12.3) | 326 (14.9) | 8.618a | 0.003 |
| NO | 4021 (87.7) | 1859 (85.1) |  |  |
| **Physical labor before, n (%)** |  | |  | |
| YES | 3495 (77.4) | 1580 (74.1) | 8.642a | 0.003 |
| NO | 1019 (22.6) | 551 (25.9) |  |  |
| **Exercise, n (%)** |  | |  | |
| YES | 1698 (36.4) | 437 (19.7) | 193.277a | <0.001 |
| NO | 2959 (63.6) | 1776 (80.3) |  |  |
| **Activities, n (%)** |  | |  | |
| YES | 4183(90) | 1213 (54.8) | 1103.517a | <0.001 |
| NO | 466 (10) | 1000 (45.2) |  |  |
| **Tai chi, n (%)** |  |  |  |  |
| YES | 201 (4.3) | 52 (2.3) | 16.448a | <0.001 |
| NO | 4448 (95.7) | 2161 (97.7) |  |  |
| **Square dance, n (%)** |  | | | |
| YES | 352 (7.6) | 66 (3) | 55.198a | <0.001 |
| NO | 4297 (92.4) | 2147 (97) |  |  |
| **Garden work, n (%)** |  | | | |
| YES | 1217 (26.2) | 257 (11.6) | 188.563a | <0.001 |
| NO | 3432 (73.8) | 1956 (88.4) |  |  |
| **Raising animals/pets, n (%)** |  | | | |
| YES | 1445 (31.1) | 304 (13.7) | 237.505a | <0.001 |
| NO | 3204 (68.9) | 1909 (86.3) |  |  |
| **playing cards/mah-jongg, n (%)** |  | | | |
| YES | 1170 (25.2) | 244 (11) | 183.258a | <0.001 |
| NO | 3749 (74.8) | 1969 (89) |  |  |
| **Social activity, n (%)** |  | | | |
| YES | 927 (19.9) | 200 (9) | 129.828a | <0.001 |
| NO | 3722 (80.1) | 2013 (91) |  |  |
| **Housework, n (%)** |  | | | |
| YES | 3605 (77.5) | 885 (40) | 934.782a | <0.001 |
| NO | 1044 (22.5) | 1328 (60) |  |  |

**Supplementary Table S2. Multiple logistic regression between exercise/activities and frailty based on gender.**

|  | **Male** | | | | **Female** | | | |
| --- | --- | --- | --- | --- | --- | --- | --- | --- |
|  | **Frailty *OR*（95%*CI*）** | ***P*** | **Adjust frailty *OR*（95%*CI*）** | ***P*** | **Frailty *OR*（95%*CI*）** | ***P*** | **Adjust frailty *OR*（95%*CI*）** | ***P*** |
| **Exercise** |  | | | |  | | | |
| YES | Reference |  | Reference |  | Reference |  | Reference |  |
| NO | 0.56(0.46－0.68) | <0.001 | 1.93(1.51－2.45) | <0.001 | 0.53(0.44－0.63) | <0.001 | 1.85(1.48－2.32) | <0.001 |
| **Activities** |  | | | |  | | | |
| YES | Reference |  | Reference |  | Reference |  | Reference |  |
| NO | 6.81(5.68－8.16) | <0.001 | 4.31(3.43－5.42) | <0.001 | 8.68(7.13－10.57) | <0.001 | 4.68(3.61－6.07) | <0.001 |
| **Age (years)** |  | | 1.10(1.08－1.11) | <0.001 |  | | 1.10(1.07－1.09) | <0.001 |
| **Residence** |  | | | |  | | | |
| city |  |  | Reference |  |  |  | Reference |  |
| Town |  |  | 0.81(0.59－1.11) | 0.200 |  |  | 0.78(0.59－1.02) | 0.072 |
| Rural |  |  | 0.75(0.54－1.02) | 0.074 |  |  | 0.72(0.55－0.95) | 0.019 |
| **Years of schooling (years)** |  | | | |  | | | |
| 0 |  |  | Reference |  |  |  | Reference |  |
| 1-6 |  |  | 0.79(0.61－1.029) | 0.081 |  |  | 0.73(0.58－0.91) | 0.006 |
| 7-9 |  |  | 0.81(0.56－1.16) | 0.251 |  |  | 0.97(0.65－1.46) | 0.895 |
| 10-12 |  |  | 0.59(0.37－0.94) | 0.026 |  |  | 1.01(0.54－1.89) | 0.980 |
| At lest 13 |  |  | 1.04(0.60－1.81) | 0.879 |  |  | 1.44(0.70－2.98) | 0.319 |
| **Co-residence** |  | | | |  | | | |
| Living with housemember(s) |  |  | Reference |  |  |  | Reference |  |
| Living alone |  |  | 0.95(0.70－1.28) | 0.720 |  |  | 0.89(0.71－1.12) | 0.327 |
| In an institution |  |  | 1.54(0.81－2.92) | 0.190 |  |  | 1.65(0.96－2.85) | 0.072 |
| **Economic situation** |  | | | |  | | | |
| Very rich |  |  | Reference |  |  |  | Reference |  |
| rich |  |  | 0.77(0.41－1.45) | 0.415 |  |  | 0.80(0.40－1.59) | 0.521 |
| So so |  |  | 1.08(0.59－1.96) | 0.804 |  |  | 1.35(0.70－2.62) | 0.368 |
| poor |  |  | 3.66(1.87－7.16) | <0.001 |  |  | 2.45(1.19－5.05) | 0.015 |
| Very poor |  |  | 3.37(1.27－8.98) | 0.015 |  |  | 2.68(1.06－6.83) | 0.038 |
| **Smoking** |  | | | |  | | | |
| Yes |  |  | Reference |  |  |  | Reference |  |
| No |  |  | 1.14(0.89－1.46) | 0.297 |  |  | 0.81(0.52－1.26) | 0.352 |
| **Drinking** |  | | | |  | | | |
| Yes |  |  | Reference |  |  |  | Reference |  |
| No |  |  | 1.40(1.08－1.83) | 0.013 |  |  | 1.81(1.20－2.72) | 0.005 |
| **BMI (kg/m^2^)** |  |  | 1.01(0.10－1.02) | 0.149 |  |  | 1.01(1.00－1.03) | 0.041 |
| **Heart disease** |  | | | | | | | |
| Yes |  |  | Reference |  |  |  | Reference |  |
| No |  |  | 0.69(0.51－0.93) | 0.015 |  |  | 0.80(0.63－1.01) | 0.058 |
| **Stroke or CVD** |  | | | | | | | |
| Yes |  |  | Reference |  |  |  | Reference |  |
| No |  |  | 0.87(0.60－1.27) | 0.464 |  |  | 0.77(0.59－1.03) | 0.074 |
| **Nutrient supplements** |  | | | |  | | | |
| YES |  |  | Reference |  |  |  | Reference |  |
| NO |  |  | 0.69(0.50－0.95) | 0.022 |  |  | 0.58(0.45－0.75) | <0.001 |
| **Physical labor before** |  | | | |  | | | |
| YES |  |  | Reference |  |  |  | Reference |  |
| NO |  |  | 1.38(1.05－1.82) | 0.020 |  |  | 0.95(0.75－1.21) | 0.698 |

**Supplementary table S3. The association between activities and frailty**

| **Ref=yes** | **Frailty *OR*（95%*CI*）** | ***P* value** | **Adjust frailty *OR*（95%*CI*）** | ***P* value** |
| --- | --- | --- | --- | --- |
| **Tai chi** | 0.95(0.67－1.35) | 0.785 | 0.85(0.56－1.31) | 0.464 |
| **Square dance** | 1.80(1.34－2.42) | <0.001 | 1.67(1.16－2.40) | 0.006 |
| **Garden work** | 1.78(1.52－2.08) | <0.001 | 1.35(1.12－1.64) | 0.002 |
| **Raising animals/pets** | 1.91(1.65－2.22) | <0.001 | 1.48(1.22－1.78) | <0.001 |
| **playing cards/mah-jongg** | 2.20(1.88－2.58) | <0.001 | 1.77(1.45－2.16) | <0.001 |
| **Social activity** | 1.78(1.49－2.13) | <0.001 | 1.52(1.22－1.89) | <0.001 |
| **Housework** | 4.09(3.65－4.59) | <0.001 | 3.47(2.96－4.07) | <0.001 |

**Supplementary table S4. The association between frequency of activities and frailty**

| **(ref=more than once a week)** | **Frailty *OR*（95%*CI*）** | ***P* value** | **Adjust frailty *OR*（95%*CI*）** | ***P* value** |
| --- | --- | --- | --- | --- |
| **Tai chi** | 1.12(0.75－1.67) | 0.585 | 0.93(0.57－1.51) | 0.768 |
| **Square dance** | 1.87(1.32－2.66) | <0.001 | 1.74(1.12－2.69) | 0.014 |
| **Garden work** | 1.87(1.58－2.20) | <0.001 | 1.36(1.11－1.66) | 0.003 |
| **Raising animals/pets** | 1.93(1.66－2.25) | <0.001 | 1.47(1.21－1.79) | <0.001 |
| **playing cards/mah-jongg** | 2.31(1.91－2.79) | <0.001 | 1.86(1.46－2.35) | <0.001 |
| **Social activity** | 1.80(1.37－2.36) | <0.001 | 1.33(0.96－1.85) | 0.083 |
| **Housework** | 3.89(3.47－4.35) | <0.001 | 3.45(2.95－4.04) | <0.001 |

**Supplementary Table S5. The association between the types of activities carried out simultaneously and frailty**

| **Number of Activities** | **Frailty *OR*（95%*CI*）** | ***P* value** | **Adjust frailty *OR*（95%*CI*）** | ***P* value** |
| --- | --- | --- | --- | --- |
| **0 (Reference)** | 1.00 |  | 1.00 |  |
| **1** | 0.23(0.20－0.27) | <0.001 | 0.30(0.25－0.36) | <0.001 |
| **2** | 0.11(0.10－0.13) | <0.001 | 0.18(0.14－0.22) | <0.001 |
| **3** | 0.06(0.05－0.08) | <0.001 | 0.13(0.10－0.17) | <0.001 |
| **4** | 0.05(0.04－0.08) | <0.001 | 0.11(0.07－0.17) | <0.001 |
| **5** | 0.04(0.02－0.09) | <0.001 | 0.11(0.05－0.24) | <0.001 |
| **6** | 0.13(0.04－0.41) | <0.001 | 0.30(0.09－1.04) | 0.058 |
| **7** | 0.23(0.07－0.78) | 0.018 | 0.51(0.12－2.17) | 0.362 |

This table presents the odds ratios (OR) and adjusted odds ratios (AOR) for frailty associated with the number of activities carried out simultaneously among older adults. The reference group is set to "0 activities," representing individuals who do not engage in any of the specified activities. The adjusted odds ratios account for potential confounders such as age, gender, BMI, chronic diseases, and socioeconomic status.

**Value assignment**

| **Frailty** | Yes=1 | No=0 |
| --- | --- | --- |
| **Gender** | Male=1 | Female=2 |
| **Residence** | City=1; Town=2; Rural=3 | |
| **Years of schooling** | 0=1; 1-6=2; 7-9=3; 10-12=4; 13=5 | |
| **Co-residence** | Living with housemember(s)=1  Alone=2  Situation=3 | |
| **Economic situation** | Very rich=1; rich=2; so-so=3  poor=4; very poor=5 | |
| **Smoking** | Yes=1 | No=2 |
| **Drinking** | Yes=1 | No=2 |
| **Labor work before** | Yes=1 | No=2 |
| **BMI (kg/m^2^)** |  | |
| **Diabetes** | Yes=1 | No=2 |
| **Heart disease** | Yes=1 | No=2 |
| **Stroke or CVD** | Yes=1 | No=2 |
| **Nutrient supplement** | Yes=1 | No=2 |
| **Exercise** | Yes=1 | No=2 |
| **Activities** |  | |
| **Tai chi-quan** | Yes=1 | No=2 |
| **Square dance** | Yes=1 | No=2 |
| **Garden work** | Yes=1 | No=2 |
| **Raising animals/pets** | Yes=1 | No=2 |
| **playing cards/mah-jongg** | Yes=1 | No=2 |
| **Social activity** | Yes=1 | No=2 |
| **Housework** | Yes=1 | No=2 |
| **Frequency of activities** | 1=at least once a week; 2=less than once a week | |
| **Simultaneous activities** | 0=0 ; 1=1 ; 2=2 ; 3=3; 4=4; 5=5; 6=6; 7=7 | |

**APPENDIX**

|  | Variable | Classification |
| --- | --- | --- |
| 1 | Feeding | With assistance=1，without assistance=0 |
| 2 | Bathing | With assistance=1，without assistance=0 |
| 3 | Dressing | With assistance=1，without assistance=0 |
| 4 | Toileting | With assistance=1，without assistance=0 |
| 5 | Indoor Transferring | With assistance=1，without assistance=0 |
| 6 | Continence | With assistance=1，without assistance=0 |
| 7 | Able to go outside to visit neighbors | Difficult or unable=1，Yes=0 |
| 8 | Able to go shopping | Difficult or unable=1，Yes=0 |
| 9 | Able to make food | Difficult or unable=1，Yes=0 |
| 10 | Able to wash clothes | Difficult or unable=1，Yes=0 |
| 11 | Able to walk one kilometer | Difficult or unable=1，Yes=0 |
| 12 | Able to carry 5kg weight | Difficult or unable=1，Yes=0 |
| 13 | Able to crouch and stand three times | Difficult or unable=1，Yes=0 |
| 14 | Able to take public transportation | Difficult or unable=1，Yes=0 |
| 15 | Hypertension | Yes=1，No=0 |
| 16 | Diabetes | Yes=1，No=0 |
| 17 | Heart disease | Yes=1，No=0 |
| 18 | Stroke or CVD | Yes=1，No=0 |
| 19 | Bronchitis, emphysema, pneumonia, asthma | Yes=1，No=0 |
| 20 | Cancer | Yes=1，No=0 |
| 21 | Arthritis | Yes=1，No=0 |
| 22 | MMSE | <10=1，11-17=0.75，18-20=0.5，20-24=0.25，>24=0 |
| 23 | Visual function | Can't see or blind=1，only see=0.5，can see and distinguish=0 |
| 24 | Self-reported health | Bad or very bad=1，so so=0.5，good or very good=0 |
| 25 | Change of health since last year | Worse =1，Better/Same =0 |
| 26 | Look on the bright side of things | Seldom or never=1，sometimes=0.5，Always or often=0 |
| 27 | Feel useless and hard work with age | Always or often=1，sometimes=0.5，Seldom or never=0 |
| 28 | Feel lonely | Always or often=1，sometimes=0.5，Seldom or never=0 |
| 29 | Feel happy as young | Seldom or never=1，sometimes=0.5，Always or often=0 |
| 30 | Able to stand up from sitting | yes, without using hands=0，yes, using hands=0.5，no=1 |
| 31 | Hand behind neck | neither hand=1，one hand=0.5，both hands=0 |
| 32 | Hand behind lower back | neither hand=1，one hand=0.5，both hands=0 |
| 33 | Sleep quality | Bad or very bad=1，so so=0.5，good or very good=0 |
| 34 | BMI | ＜18.5 or ≥30=1，25-30=0.5，18.5-25=0 |
| 35 | Feel sad or depressed | Always or often=1，sometimes=0.5，Seldom or never=0 |
| 36 | Difficulty with hearing | Yes=1，No=0 |
| 37 | Keep things clean and tidy | Seldom or never=1，sometimes=0.5，Always or often=0 |
| 38 | Limited in activities because of health problem for the last 6 months | Yes=1，No=0 |
